# ADAMTS7 Enhances Gastric Cancer Growth and Metastasis by Triggering the NF-κB Signaling Pathway

**DOI:** 10.7150/jca.103093

**Published:** 2025-01-01

**Authors:** Shun Chen, Jiancheng He, Hanxu Gao, Xian Gao, Lingchen Dai, Junjie Chen, Zhenyu Sha

**Affiliations:** 1 Department of Gastrointestinal Surgery, Affiliated Hospital and Medical School of Nantong University, Nantong 226001, China.; 2 Research Center of Clinical Medicine, Affiliated Hospital of Nantong University, Nantong, 226001, China.; 3 Nantong Key Laboratory of Gastrointestinal Oncology, Nantong, 226001, China.; 4 High Quality Development Assessment Office, Affiliated Hospital of Nantong University, No 20, Xisi Road, Nantong 226001, China.

**Keywords:** ADAMTS7, Gastric cancer, NF-κB, Metastasis, Proliferation

## Abstract

The ADAMTS (a disintegrin and metalloproteinase with thrombospondin motifs) family of metalloproteinases plays a vital role in various biological and pathological processes, including tissue remodeling, angiogenesis, and cancer progression. Among the 19 ADAMTS family members, our research focused on ADAMTS7, which exhibited significant overexpression in gastric cancer (GC). This overexpression was strongly correlated with poor clinical outcomes, including reduced overall survival and heightened metastatic potential. To investigate the role of ADAMTS7 in GC, we employed an integrated approach encompassing bioinformatics analysis, Western blotting, immunofluorescence, as well as *in vitro* and *in vivo* functional analyses. Our results showed that silencing ADAMTS7 expression significantly inhibited the proliferation, migration, and invasion of GC cells, and furthermore, silencing ADAMTS7 significantly inhibited the growth and metastasis of tumour cells *in vivo* in nude mice, highlighting its critical role in driving the malignant behaviour of GC cells. Further mechanistic studies identified the NF-κB signaling pathway as a key downstream target of ADAMTS7, with ADAMTS7 silencing resulting in a notable reduction in NF-κB pathway activity. These findings establish ADAMTS7 as a significant contributor to the aggressiveness of GC and a pivotal activator of the NF-κB pathway, a major regulator of inflammation and tumor progression. Consequently, ADAMTS7 emerges as a promising therapeutic target and prognostic biomarker for GC. Our study opens new avenues for the development of targeted therapies aimed at inhibiting ADAMTS7 activity, thereby potentially improving treatment outcomes and survival rates for patients with GC.

## Introduction

GC is a major and widespread form of cancer in the gastrointestinal tract. The disease significantly impacts the quality of life and health of patients and is a leading cause of cancer mortality worldwide^1^, ^2^. Despite recent advancements in diagnostic and therapeutic techniques, the prevalence and mortality rates of GC in the general population have been observed to increase^3^, ^4^, presenting a considerable challenge for patients and the healthcare system^5^. GC is highly aggressive, and its development and progression are closely related to various complex factors and mechanisms, including genetic background, environmental influences, lifestyle, and microbial infections. These factors collectively pose significant challenges to the prevention and treatment of GC^6^. Comprehensive research into the etiology of GC and the identification of potential therapeutic targets are imperative to developing novel therapeutic modalities and markedly improving the prognosis and quality of life for patients^7^-^9^.

The ADAMTS family is a diverse group of metalloproteinases known for their roles in various biological processes. Comprising 19 secreted zinc-dependent enzymes, ADAMTS proteases possess a unique N-terminal region composed of a pre-propeptide domain, a metalloproteinase domain, and a disintegrin-like domain. These enzymes primarily target extracellular matrix (ECM) components, playing a pivotal role in tissue remodeling and the maintenance of homeostasis through their capacity to degrade these components^10^, ^11^. Dysregulation of ADAMTS protein expression or function promotes tumorigenic and angiogenic activity^12^. In GC, there is a notable correlation between reduced ADAMTS9 expression and an increased likelihood of lymphatic metastasis, which may portend a poor prognosis for patients^13^. Additionally, as tumor suppressors, low expression of ADAMTS8 and ADAMTS19 is significantly correlated with tumor invasion depth and metastasis^14^, ^15^. Conversely, ADAMTS12 has been identified as an important driver of GC chemoresistance and poor prognosis^16^. The upregulation of ADAMTS2 and ADAMTS16 is significantly associated with an unfavorable prognosis in GC patients^17^, ^18^. However, despite some members of the ADAMTS family attracting attention in GC development, the specific roles and mechanisms of other members remain poorly understood. Therefore, further studies are essential to elucidate the potential roles of these ADAMTS family members in the GC process.

Dysregulated activation of NF-κB represents a significant contributing factor in the pathogenesis of GC^19^. The NF-κB signaling pathway is a classical pathway activated by numerous external stimuli and upstream signals. The process initiates the NF-κB signaling pathway when the IκB kinase complex phosphorylates IκBα^20^-^22^. Consequently, p65 translocates to the nucleus, where it binds to the p65 response elements, thereby promoting specific gene transcription^23^. The NF-κB signaling pathway, a regulator of inflammation^23^-^25^, also provides resistance to apoptosis and genotoxic damage, promoting tumor growth and angiogenesis through the production of growth factors^26^, ^27^. A substantial body of evidence from scientific studies indicates that NF-κB plays a pivotal role in the initiation and progression of numerous tumors. The NF-κB pathway regulates several essential biological processes, including cell apoptosis, growth, and differentiation. Moreover, it has been demonstrated to influence the efficacy and toxicity of chemotherapy and radiotherapy in tumor cells^28^-^31^. However, the specific mechanisms of NF-κB signaling in regulating GC development remain unclear and warrant further exploration.

The present study identified a significant upregulation of ADAMTS7 expression among members of the ADAMTS family in GC tissues. Further analysis revealed that ADAMTS7 expression is associated with a poor prognosis in GC patients, suggesting its potential role in GC progression and outcomes. Experiments showed that silencing the ADAMTS7 gene significantly inhibited tumor cell proliferation and metastasis. Our findings indicate that ADAMTS7 regulates these processes through the NF-κB signaling pathway.

## Materials and methods

### Patients

A total of 120 cases of human GC tissues and their paired normal tissues were collected from the Affiliated Hospital of Nantong University between 2014 and 2016. Detailed clinical data of GC patients are shown in Table 1. Follow-up of the patients ended in August 2021 (median follow-up 53 months; range, 3-79 months). All specimens were immediately stored at -80 °C and collected at the time of surgical resection. Between 2022 and 2023, 20 pairs of GC samples and their corresponding non-cancerous samples were obtained from the same collection point for quantitative real-time PCR (qRT-PCR) analysis. Each GC case was independently confirmed by two pathologists through pathological evaluation. None of the patients received any form of adjuvant therapy before undergoing radical surgery. The study was approved by the Ethics Committee of the Affiliated Hospital of Nantong University, and all patients provided written informed consent before participating in the study. (2023-K076-01).

### Cell culture and reagents

Normal gastric mucosal epithelial cells (GES-1) and GC cell lines SGC-7901 and BGC-823 were sourced from Genechem (Shanghai, China). The GC cell line MKN-45 was sourced from the BeNa Culture Collection (Shanghai, China), while HGC-27 and AGS were obtained from the Cell Bank of the Chinese Academy of Sciences (Shanghai, China). Cells were incubated in RPMI-1640 medium, supplemented with 10% fetal bovine serum and penicillin-streptomycin, in a humidified atmosphere at 37 °C with 5% CO₂, in accordance with standard laboratory practices. DAPI was obtained from Cell Signaling Technology (Boston, USA). HY-134476 was purchased from MedChemExpress (New Jersey, USA). For the drug treatment group, a concentration of 1 μM HY-134476 was applied to cells within the experimental group for a period of 6 hours^32^. Each experiment was conducted in triplicate.

### Cell transfection

An ADAMTS7-targeting shRNA was designed and synthesised by Tsingke (Beijing, China). sh- ADAMTS7 virus were purchased from RiboBio (Guangzhou, China). The sequence is as follows:

Negative control: UUCUUCGAAGGUGUCACGUTT;

ADAMTS7sh-#1: CGCCTTCTACGAGCTACAATA;

ADAMTS7sh-#2: ACCCTCCTGCCAAGGACATTA;

ADAMTS7sh-#3: TTCTGCGAGGACATGGATAAT;

ADAMTS7sh-#4: CCCGTGTTCTCCTGGCATTAT;

ADAMTS7sh-#5: ACTTCTACTACGACTACAATT;

Plasmid and shRNA transfections were performed in 6-well plates using Polyplus jetPRIME transfection reagent (Polyplus, France) as per the manufacturer's instructions. GC cells were seeded into 6-well plates, the virus concentration was adjusted at MOI=100 before the medium was added, and finally 10 μg/ml polybrene was added for virus transfection. The plates were gently shaken to ensure uniform distribution and left to incubate overnight at 37 ℃. The culture medium was renewed 24 hours later, and transfection efficiency was assessed 48 hours later.

### qRT-PCR assay

Total RNA from gastric carcinoma tissues and cell cultures was isolated using the Trizol method, as recommended by the supplier (Invitrogen, USA), according to the instructions provided^33^. cDNA was synthesised using the cDNA Synthesis Kit (Roche) according to the manufacturer's procedures. Real-time PCR was performed on an ABI PCR system using the SYBR Green I Master Kit (Roche, China) with the following primer sequences:

ADAMTS7-forward: 5'-GTCATCGACTTCCCTTCCATAC-3 ';

ADAMTS7-reverse: 5'-TGTCCATGTCATCGCAGAAG-3 ';

GAPDH-forward: 5'-AGAAGGCTGGGGGCTCA TTTG-3 ';

GAPDH-reverse: 5'-AGGGGCCATCCACAGTCTTC-3 ';

GAPDH was used as internal reference. All primers were purchased from Tsingke Company, Beijing, China.

### Western blot assay

Western blotting was carried out according to the previously mentioned protocol^34^, ^35^. The antibodies used included: anti-p-IKKβ (Cell Signaling Technology, USA), anti-p-IKBα, anti-ADAMTS7, anti-GAPDH, anti-LaminB1, and anti-NF-κB p65 (Proteintech, Wuhan, China).

### Immunofluorescence assay (IF)

IF was conducted as outlined in our prior research^34^, ^36^. Treated GC cells were washed with PBS. Cells were then incubated with anti-NF-κB (1:500, Proteintech, Wuhan, China) at 4 °C overnight, washed with PBS and stained with secondary antibody (ABclonal) at 37 °C for 2 hours. The nuclei were stained with DAPI for 10 minutes. Stained cell images were captured using a Zeiss LSM 900 confocal microscope. All experiments were performed in triplicate.

### Tissue Microarray (TMA) and Immunohistochemistry (IHC)

TMA and IHC were conducted following previously established methods^37^. The following antibodies were employed in the study: anti-ADAMTS7, anti-Ki67, anti-NF-κB p65. The staining intensity was manually evaluated by two experienced pathologists using the following criteria: 0 (negative), 1 (weak positive), 2 (moderate positive), 3 (strong positive). The score was then based on the positive rate:0(<5%) 1 (5-25%), 2 (>25-50%), 3 (>50-75%), 4 (>75%). The final IHC score was calculated as the product of the intensity score and the staining proportion. High expression was defined as a score ≥3, while low expression was defined as a score ≤2^34^, ^38^-^40^.

### Transwell and wound healing assays

Transwell and wound healing assays were performed following established protocols^41^.

### Cell proliferation and colony formation assay

The DNA synthesis rate was determined using the Click-iT EDU Imaging Kit (Beyotime, Beijing, China) following the manufacturer's instructions. For CCK-8 assay, 10 µL CCK-8 solution (Dojindo, Kumamoto, Japan) was added to 96-well plates according to directions. After incubating 2×10^3^ cells for 2 hours at 37 ℃, absorbance was read at 450 nm. For cloning experiments, cell colonies were stained with crystal violet and incubated in 6-well plates for 14 days. All experiments were conducted in triplicate.

### Bioinformatics analysis

Expression and prognostic significance of ADAMTS family genes were analysed using The Cancer Genome Atlas (TCGA) database with GEO microarrays (GSE65801, GSE51575, GSE33335, GSE30727). The expression levels of the ADAMTS family genes were obtained. They were divided into two groups: one comprising tumour samples and the other comprising normal samples. The Mann-Whitney U test was used to assess statistical differences in expression levels between the two groups. ADAMTS7 expression was categorized into high and low groups based on the average value. Differential analysis was conducted with the R language (edgeR package), and Gene Set Enrichment Analysis (GSEA) utilized the gene set h.all.v2023.1.Hs.symbols.gmt.

### Animal experimentation

BALB/c nude mice (male, six weeks old) were obtained from the Animal Testing Center at Nantong University (Nantong, China). Establishment of an intraperitoneal implantation metastasis model in nude mice GC cells transfected with Sh-ADAMTS7 or Sh-NC, 6 × 10^^6^ GC cells were injected intraperitoneally into nude mice (5 nude mice per group). After 30 days, mice were humanely euthanised and their nodules were dissected and counted. For tumour formation experiments, 6 × 10^6 GC cells transfected with Sh-ADAMTS7-containing cells or Sh-NC-containing cells were injected subcutaneously into the upper forelimb (A total of five nude mice were included in each experimental group). Tumour volume was quantified at three-day intervals, employing the formula V = 0.5 × length × width². 24 days post-injection, nude mice were humanely killed, dissected, weighed and immunohistochemically analysed. The Animal Experimentation and Ethics Committee of Nantong University approved all animal-related studies, and procedures followed contemporary standards for animal welfare and care.

### Statistical analysis

Data analysis utilized GraphPad Prism 8.0 and Rstudio (v4.2.3). Measurement data with normal distribution were expressed as mean ± SD and statistically analyzed with two-sample t test. Quantitative data that are not normally distributed are presented as the median (range) and are analysed using the Mann-Whitney U test. The data was analysed via the chi-square test, with categorical variables presented as case numbers (percentage). For comparisons between groups of normally distributed data, one-way analysis of variance (ANOVA) was used. The Kruskal-Wallis test was applied for group comparisons involving non-normally distributed data. Survival rates in gastric cancer (GC) patients were analyzed using Kaplan-Meier curves and the log-rank test, while prognostic factors were identified through COX regression analysis. The statistical analyses were conducted with a two-sided approach and a level of statistical significance of P < 0.05. All experiments were conducted at least three times.

## Results

### Analysis of ADAMTS7 expression and prognosis in the database

**Figure 1:**
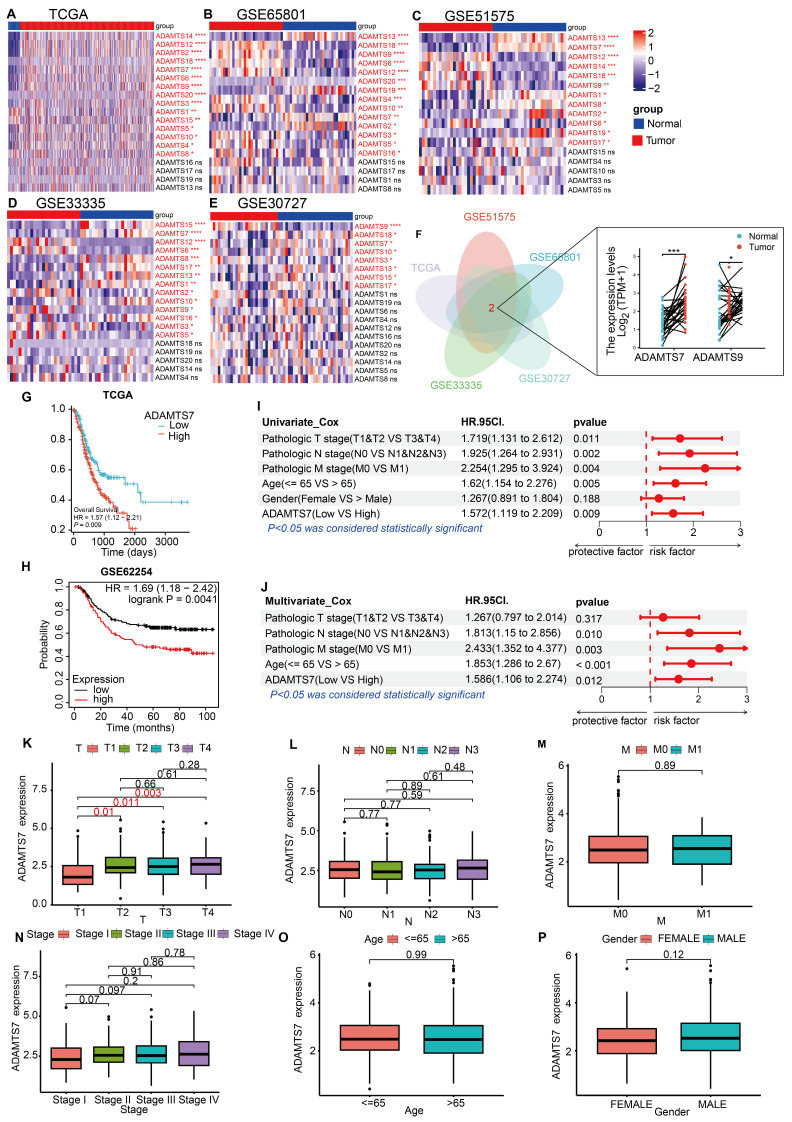
** Analysis of ADAMTS7 expression and prognosis in the database. A-D** Differential expression of ADAMTS member genes in TCGA database with GSE65801, GSE51575, GSE33335, GSE30727. *P < 0.05; **P < 0.001; ***P < 0.0001; ****P < 0.00001. **F** Wayne plots using differential genes for TCGA, GSE65801, GSE51575, GSE33335, GSE30727. *P < 0.05; ***P < 0.001.**G** OS in patients with high or low expression of ADAMTS7 in GC was analysed using the Kaplan-Meier method. **H** Analysis of ADAMTS7 expression in relation to OS in patients with GC based on the Kaplan-Meier Plotter database. **I-J** The TCGA dataset was subjected to both single-factor and multifactor Cox regression analyses. **K-P** Relationship between ADAMTS7 expression and clinicopathological parameter.

To identify GC-related ADAMTS members, we first screened the TCGA database and four GEO microarrays (GSE65801, GSE51575, GSE33335, GSE30727) for differentially expressed ADAMTS genes. Results showed that ADAMTS7 and ADAMTS9 exhibit differential expression in GC (Fig. 1A-F). ADAMTS9 is a well-studied tumor suppressor in GC^13^, ^42^, ^43^. However, the role of ADAMTS7 in GC remains undefined. Therefore, we have chosen ADAMTS7 as our next research target. To determine the poor prognosis associated with ADAMTS7 in GC, the TCGA database and the Kaplan-Meier online database (http://kmplot.com/) were utilized (Fig. 1G, H).

Cox regression analyses were conducted on clinical data from the TCGA database to ascertain the prognostic significance of various factors in the context of cancer. Our findings indicate that ADAMTS7 serves as an independent risk factor (Fig. 1I, J). An examination of the connection between ADAMTS7 expression levels and the clinicopathological features of patients in the TCGA database indicated that increased ADAMTS7 expression levels were observed in GC patients with advanced T-stages. Conversely, no correlation was found between ADAMTS7 expression and the patients' gender, age, pathological stage, N stage, or M stage (Fig. 1K-P). These findings suggest that ADAMTS7 expression in GC is specifically associated with tumor invasion (T-stage) and operates independently of demographic and other clinical factors. This independence indicates that ADAMTS7 may serve as a specific marker for invasive behavior in GC, without being influenced by broader patient characteristics or disease stage, highlighting its potential clinical significance in assessing tumor aggressiveness.

### The expression of ADAMTS7 is increased in GC and is an independent risk factor for the disease

**Figure 2:**
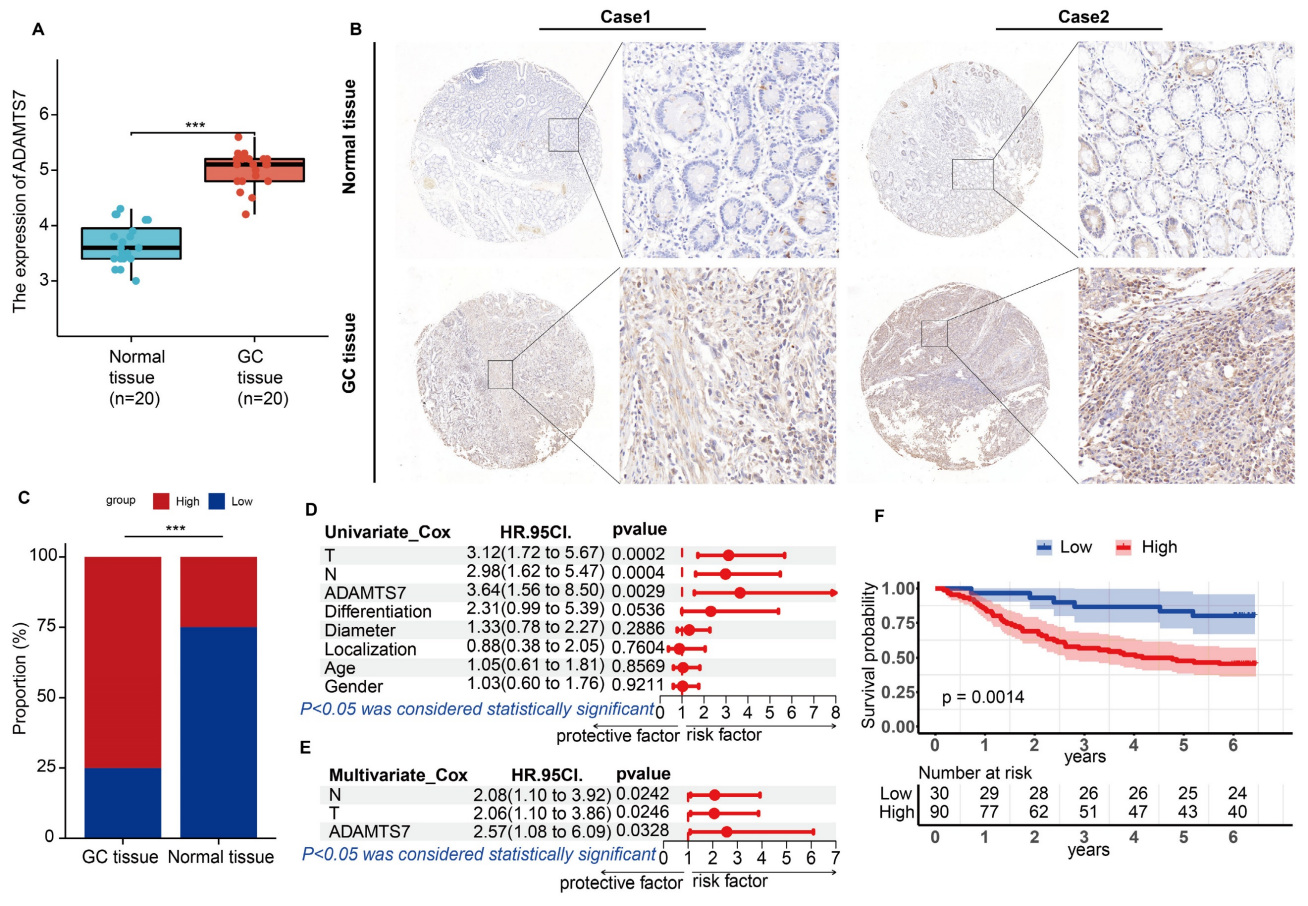
** The expression of ADAMTS7 is increased in GC and is an independent risk factor for the disease. A** ADAMTS7 mRNA expression was measured in 20 pairs of specimens. ***P < 0.001. **B** Analysis of typical tissue microarray images of ADAMTS7 expression in 120 patients. **C** Detection of ADAMTS7 protein levels in GC tissues by IHC. **D-E** 120 Clinical patients Cox regression analysis. **F** Analysis of ADAMTS7 Expression and OS in GC Patients Based on the Survival Information of 120 Clinical Patients.

The results of the qRT-PCR analysis demonstrated a notable elevation in ADAMTS7 mRNA expression levels in GC tissues relative to their matched normal counterparts (Fig. 2A). Subsequently, we conducted immunohistochemical analyses to examine the relative expression levels of the ADAMTS7 protein in GC and paracancerous tissues from 120 patients. The results demonstrated a significant elevation in ADAMTS7 expression in tumour tissues relative to paracancerous tissues (Fig. 2B, C). Subsequently, we examined the relationship between ADAMTS7 expression and a range of clinical and pathological characteristics in 120 patients. Results showed a statistically significant correlation between ADAMTS7 expression and lymph node metastasis (P = 0.011), TNM stage (P = 0.036), and infiltration depth (P = 0.001) (Table 1). Furthermore, the study found that high expression of ADAMTS7 was an independent predictor for GC patients, as determined by univariate multifactorial COX analysis of the 120 clinical samples (Fig. 2D, E). Additionally, we found that high expression of ADAMTS7 in GC patients was significantly associated with shorter overall survival (OS). This finding aligns with the predictions made by the TCGA and KM-Plotter databases (Fig. 2F). In summary, high expression of ADAMTS7 in GC is a key factor in GC progression.

### ADAMTS7 promotes the proliferation, migration and invasion of GC cells

**Figure 3:**
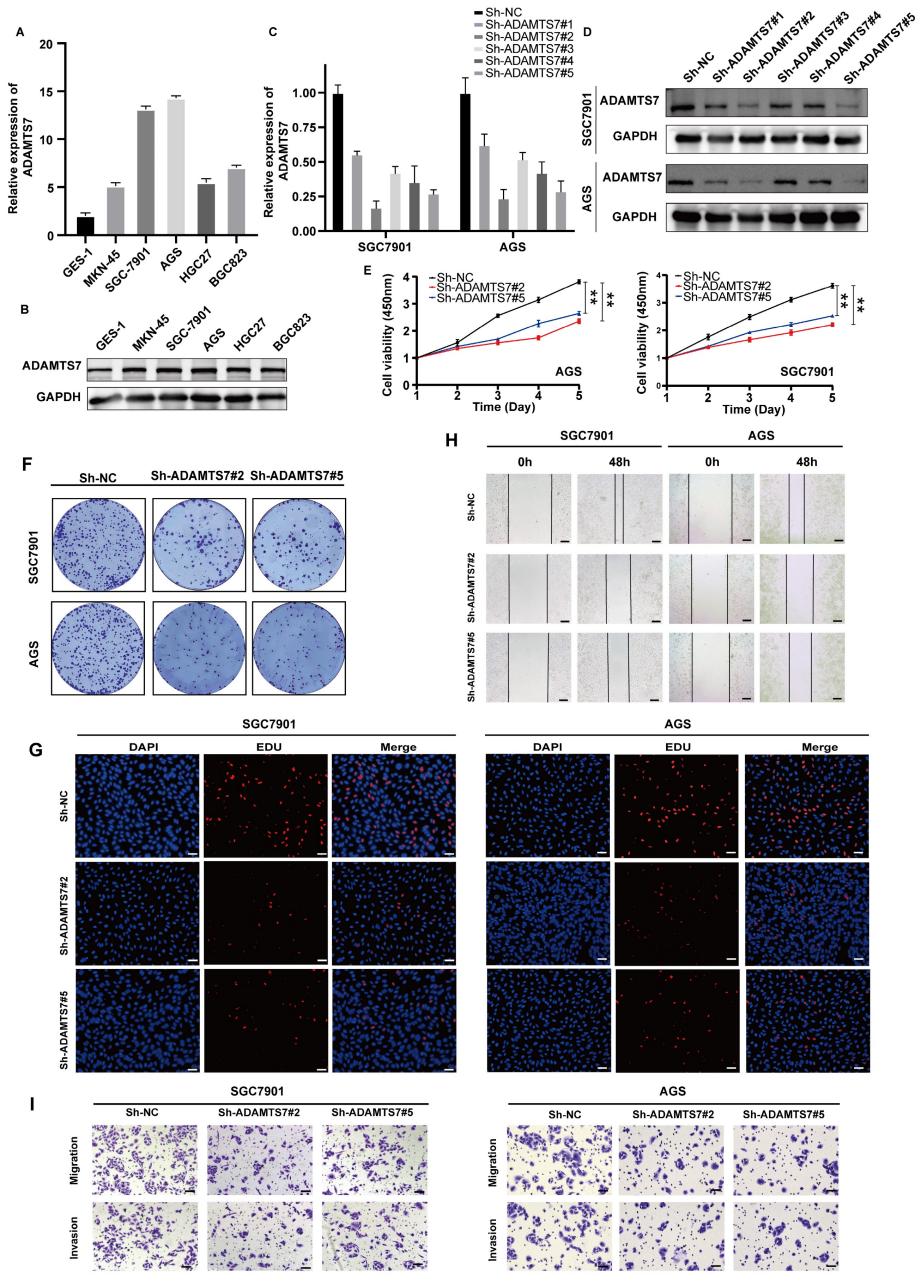
**ADAMTS7 promotes the proliferation, migration and invasion of GC cells. A-B** Expression levels of ADAMTS7 in different GC cell lines. **C-D** ADAMTS7 expression levels in SGC7901 and AGS cells transfected with Sh-NC, Sh-ADAMTS7#1, Sh-ADAMTS7#2, Sh-ADAMTS7#3, Sh-ADAMTS7#4 and Sh-ADAMTS7#5. **E-H** Cell viability was assessed using CCK-8, colony formation, and EDU assays. Scale bar, 50 μm. **I-J** Scratch assays, migration and invasion assays were used to detect cell motility. Scale bar, 20 μm. **P < 0.01; ***P < 0.001.

To investigate the role of ADAMTS7 in GC progression, we initially examined its expression in several GC cell lines, including MKN-45, AGS, HCG27, SGC-7901, and BGC823, as well as the GES-1 cell line. The findings indicated that the mRNA expression level of ADAMTS7 was significantly elevated in GC cell lines compared to GES-1 cells. Notably, SGC-7901 and AGS cells exhibited the highest expression levels (Fig. 3A, B). To explore the biological function of ADAMTS7 in GC cell lines, we knocked down ADAMTS7 in SGC-7901 and AGS cells through shRNA transfection. The transfection efficiency was confirmed by qRT-PCR and Western blot assay, which demonstrated that Sh-ADAMTS7#2 and Sh-ADAMTS7#5 markedly diminished the expression of ADAMTS7 (Fig. 3C, D). The CCK-8 assay revealed that the viability of GC cells was suppressed following the knockdown of ADAMTS7 (Fig. 3E). Similarly, the results obtained from the cloning and EDU proliferation assays indicated that knockdown of ADAMTS7 suppressed the proliferation of GC cells (Fig. 3F, G and Supplementary Fig. 1A, B). The results of the transwell and wound healing assays showed that the downregulation of ADAMTS7 inhibited the migration and invasion of GC cells compared to control cells (Fig. 3H, I and Supplementary Fig. 1C, D). These findings suggest that ADAMTS7 downregulation restrains the proliferation, migration, and invasion of GC cells.

### ADAMTS7 promotes the malignant progression of GC cells through NF-κB signalling

**Figure 4:**
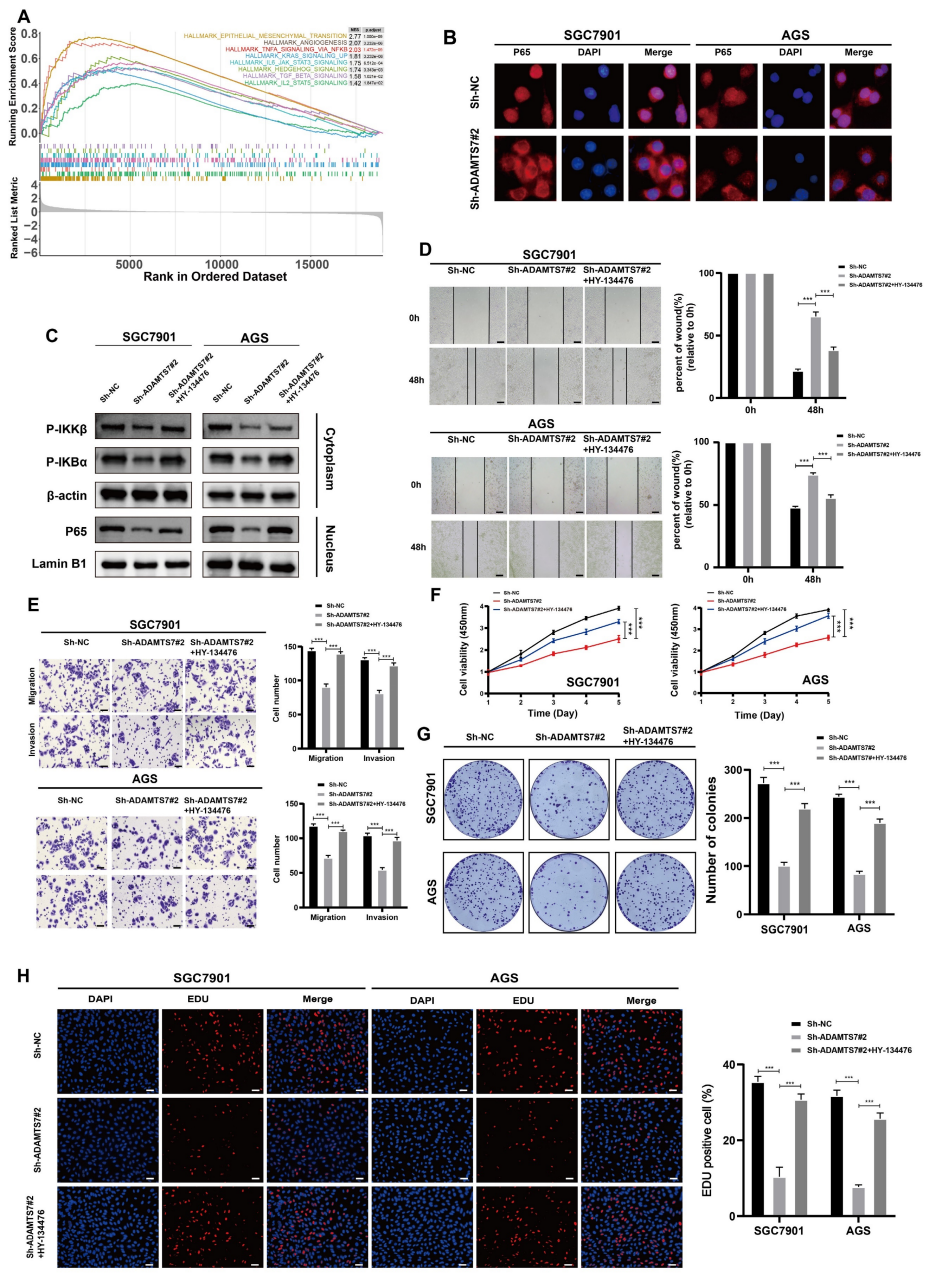
** ADAMTS7 promotes the malignant progression of GC cells through NF-κB signalling. A** GSEA analysis was conducted using the TCGA database. **B** IF assay detected activation of the NF-κB signalling pathway in SGC7901 and AGS cells. **C** Protein expression of NF-κB target genes was detected by Western blot analysis after transfection with Sh-NC, Sh-ADAMTS7#2 and Sh-ADAMTS7#2+HY-134476. **D-E** Cell motility was detected by scratch test, migration test and invasion test. Scale bar, 20 μm. **F-H** Cell viability was assessed using CCK-8, colony formation, and EDU assays. Scale bar, 50 μm. ***P < 0.001.

To elucidate the potential mechanism of ADAMTS7 in GC progression, we conducted a GSEA using the TCGA database. The results of GSEA enrichment analysis indicated that ADAMTS7 might drive the malignant progression of GC cells via the NF-κB signaling pathway (Fig. 4A). IF assay revealed a reduction in the nuclear translocation of p65 in cells with ADAMTS7 knockdown (Fig. 4B). Subsequently, we detected changes in NF-κB pathway protein levels by Western blot. It was found that ADAMTS7 knockdown suppressed the phosphorylation of IKBα and IKKβ and reduced the expression of nuclear p65 compared to control cells. Additionally, the phosphorylation levels of IKBα and IKKβ, as well as the expression of nuclear p65, were reversed after treatment of SGC-7901 and AGS (vector/sh-ADAMTS7) with the NF-κB pathway agonist HY-134476 (Fig. 4C). Transwell and scratch assays showed that the negative effect of ADAMTS7 knockdown on tumor cell migration was neutralized by the administration of HY-134476 reagents (Fig. 4D, E). CCK-8 assays demonstrated that the inhibitory effect on the viability of GC cells caused by ADAMTS7 knockdown was significantly reversed by treatment with the HY-134476 reagent (Fig. 4F). Additionally, the clonogenic assay and the EDU proliferation assay produced concordant outcomes (Fig. 4G, H). This study suggests that ADAMTS7 may contribute to the malignant behavior of GC cells through the activation of the NF-κB pathway.

### ADAMTS7 enhances the growth and metastasis of GC cells *in vivo*

**Figure 5:**
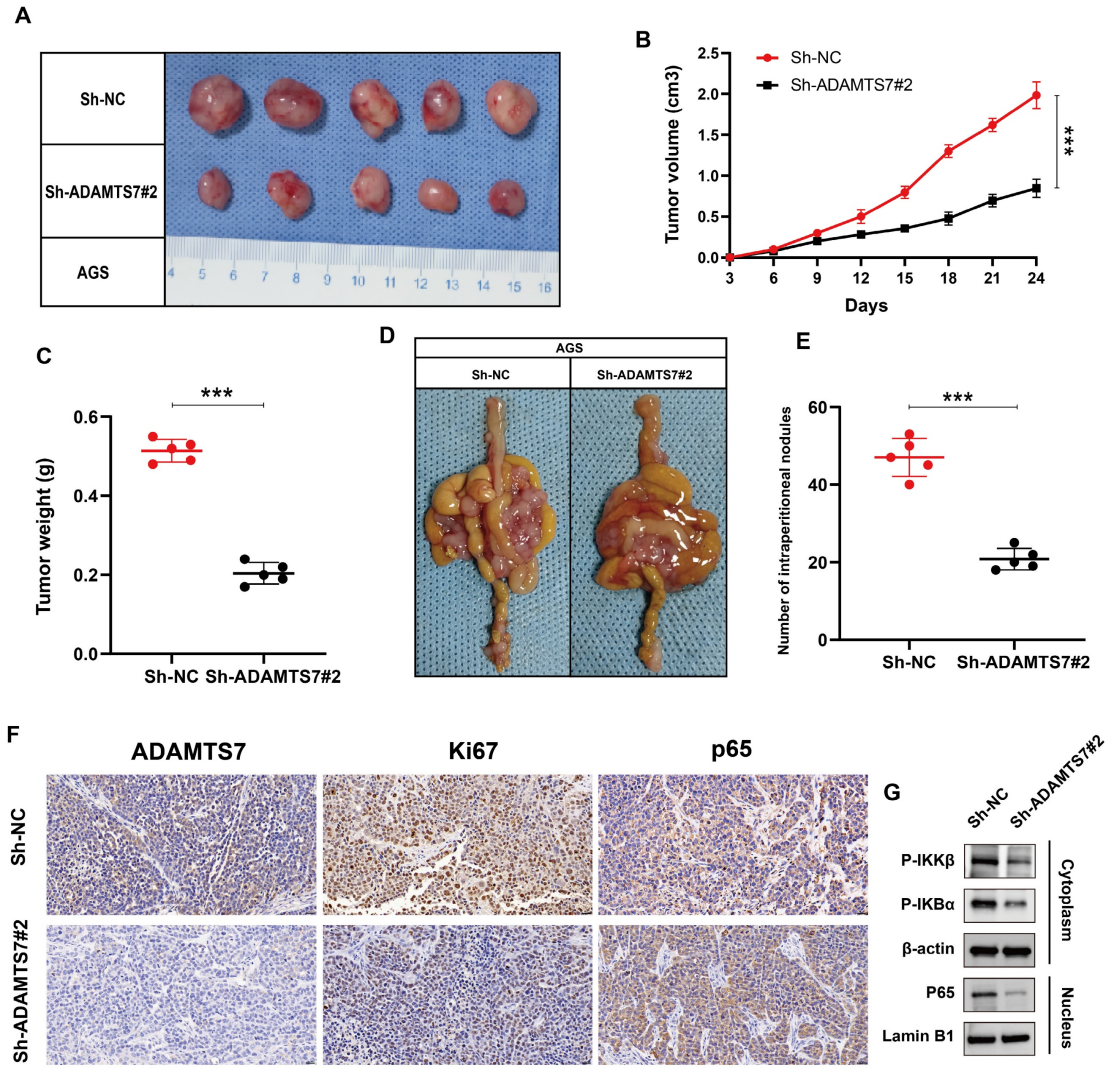
** ADAMTS7 promotes growth and metastasis of GC cells *in vivo*. A-C** Tumour growth was observed in mice following subcutaneous transplantation of treated AGS cells. The dimensions of the tumour were quantified in order to provide a visual representation of its size. **D-E** The present study presents the images and numerical data pertaining to the intraperitoneal metastatic nodules in nude mice that were injected with treated AGS cells and control cells. **F** Immunohistochemical staining for ADAMTS7, Ki67, p65. Scale bar, 20 μm. ***P < 0.001. **G** Western blot analysis of NF-κB pathway proteins in subcutaneous tumors from nude mice treated with Sh-NC and Sh-ADAMTS7#2.

To investigate whether ADAMTS7 also acts *in vivo*, we constructed xenograft tumor models using nude mice. Tumor growth in the Sh-ADAMTS7#2 group was significantly slower, with notable reductions in both size and volume over time, as well as a marked decrease in final tumor weight compared to the control group (Fig. 5A-C). In a mouse peritoneal metastasis model, the Sh-ADAMTS7#2 group showed significantly fewer intraperitoneal metastatic nodules, highlighting the role of ADAMTS7 in promoting tumor proliferation and metastasis in GC development (Fig. 5D, E). Additionally, IHC analysis of subcutaneous tumors revealed a decrease in ADAMTS7, Ki67, and nuclear p65 expression levels in the Sh-ADAMTS7#2 group, indicating reduced cell proliferation and NF-κB pathway activity (Fig. 5F). Western blot analysis further supported this, showing lowered levels of P-IKKβ and P-IκBα in the cytoplasm, along with decreased nuclear p65, thereby validating the inhibition of NF-κB signaling following ADAMTS7 knockdown (Fig. 5G). These findings collectively suggest that ADAMTS7 promotes GC cell proliferation and metastasis, likely through activation of the NF-κB signaling pathway.

## Discussion

The protein ADAMTS7, which plays a role in the regulation of blood coagulation, is encoded by the human ADAMTS7 gene located on chromosome 15. As a member of the ADAMTS protein family, ADAMTS7 exerts a pivotal influence within the ECM milieu, particularly concerning protein degradation and remodeling processes.

The ADAMTS7 protein comprises 1687 amino acids, contributing to its functional complexity and significance. Its structure includes multiple functional domains that confer roles in matrix degradation and ECM interactions. For instance, ADAMTS7 significantly contributes to tissue remodeling and disease progression in osteoarthritis through the specific degradation of the extracellular matrix protein COMP^44^. It also plays a role in arterial remodeling and affects atherosclerosis development^45^. Nevertheless, although the functions of ADAMTS family members in various tumors have been investigated, the precise molecular pathways by which ADAMTS7 contributes to tumor development remain poorly characterized, particularly in GC, our study reveals that ADAMTS7 expression is upregulated in GC and regulates the malignant behaviour of GC.

A substantial body of evidence from GC studies indicates that ADAMTS family members regulate the proliferation and metastasis of GC, primarily through their impact on specific signaling pathways. For example, in GC, overexpression of ADAMTS12 has been observed to inhibit apoptosis by promoting extracellular signal-regulated kinase (ERK) activation and augmenting the proliferation of GC cells treated with oxaliplatin. Furthermore, ADAMTS12 plays a key role in angiogenesis by upregulating ERK expression, facilitating the transcriptional expression of VEGF, and inducing chemoresistance through the MAPK/VEGF axis, thereby promoting the malignant progression of GC^16^. Additionally, downregulation of ADAMTS19 expression in GC induces nuclear aggregation of p65, affecting the transcription of S100A16, which plays a role in regulating GC progression^15^. Evidence also indicates that ADAMTS16 interacts with intracytoplasmic IκBα, resulting in its phosphorylation and degradation, activating the NF-κB signaling pathway. This activation increases the transcriptional activity of IFI27, promoting GC proliferation and metastasis^18^. The findings of this study indicate that ADAMTS7 plays a role in regulating the progression of GC via its effects on the NF-κB signaling cascade.

In conclusion, this study demonstrates that ADAMTS7 is overexpressed in GC and suggests a poor prognosis for patients. Bioinformatics analysis suggested that ADAMTS7 might influence GC cell proliferation and migration via NF-κB activation. Additionally, our experimental results validated that ADAMTS7 facilitates the malignant progression of GC by affecting p65 through its nuclear translocation. This study highlights a novel potential therapeutic target for GC, offering a new avenue for future therapeutic strategies. It is anticipated that this discovery will facilitate the advancement of more effective treatments and enhance the prognosis for patients.

## Supplementary Material

Supplementary figure.

## Figures and Tables

**Table 1:** Relationships between ADAMTS7 expression and clinicopathological characteristics of GC patients

Characteristics	High expression	Low expression	P value
n	90	30	
Gender, n (%)			0.331
male	57 (47.5%)	16 (13.3%)	
female	33 (27.5%)	14 (11.7%)	
Age, n (%)			0.589
<65	34 (28.3%)	13 (10.8%)	
≥65	56 (46.7%)	17 (14.2%)	
Depth of invasion, n (%)			< 0.001
T1+T2	34 (28.3%)	22 (18.3%)	
T3+T4	56 (46.7%)	8 (6.7%)	
Lymph node metastasis, n (%)			0.011
Negative	33 (27.5%)	19 (15.8%)	
Positive	57 (47.5%)	11 (9.2%)	
TNM stage, n (%)			0.036
I/II	64 (53.3%)	27 (22.5%)	
III	26 (21.7%)	3 (2.5%)	
Tumour diameter (cm), n (%)			0.450
<5	53 (44.2%)	20 (16.7%)	
≥5	37 (30.8%)	10 (8.3%)	
Tumour differentiation, n (%)			0.785
Poor/moderate	74 (61.7%)	24 (20%)	
Well	16 (13.3%)	6 (5%)	
Tumour location, n (%)			1.000
Down/middle	81 (67.5%)	27 (22.5%)	
Up	9 (7.5%)	3 (2.5%)	
