# Supplementary material for: ADAMTS7 Enhances Gastric Cancer Growth and Metastasis by Triggering the NF-κB Signaling Pathway
Source: J Cancer. 2025 Jan 1;16(3):1008–19. doi: 10.7150/jca.103093 (PMC11705055; doi:10.7150/jca.103093)
Supplement: Supplementary file 1 — Supplementary figure. [file jcav16p1008s1.pdf]

Supplementary Figure 1: ADAMTS7 promotes the proliferation, migration and invasion of GC cells.

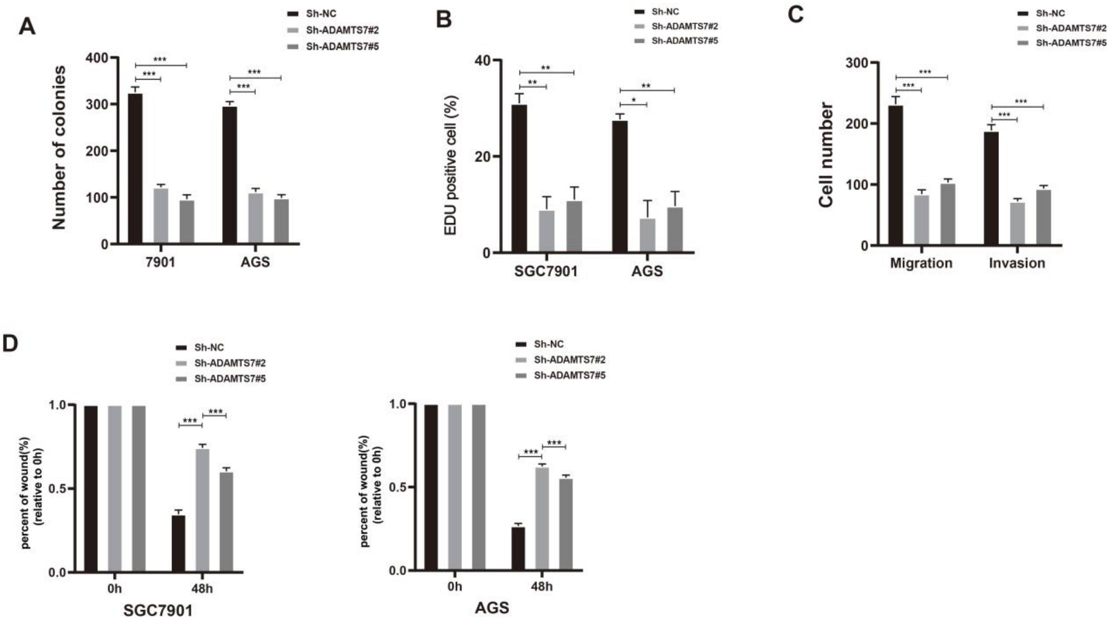

A-B Colony formation and EDU assays were performed to assess cell proliferation. **C-D** Migration, invasion, and scratch assays were used to evaluate cell motility. *P < 0.01;**P < 0.01; ***P < 0.001.
